# Potential therapeutic role of antagomiR17 for the treatment of chronic lymphocytic leukemia

**DOI:** 10.1186/s13045-014-0079-z

**Published:** 2014-10-23

**Authors:** Sara Dereani, Paolo Macor, Tiziana D’Agaro, Nelly Mezzaroba, Michele Dal-Bo, Sara Capolla, Antonella Zucchetto, Erika Tissino, Giovanni Del Poeta, Sonia Zorzet, Valter Gattei, Riccardo Bomben

**Affiliations:** Clinical and Experimental Onco-Hematology Unit, Centro di Riferimento Oncologico, I.R.C.C.S., Via Franco Gallini 2, Aviano, PN Italy; Department of Life Sciences, University of Trieste, Trieste, Italy; Division of Hematology, S.Eugenio Hospital and University of Tor Vergata, Rome, Italy

**Keywords:** CLL, MicroRNA, miR-17, AntagomiR17

## Abstract

Recently it was reported that microRNA from the *miR-17 ~ 92* family may have a key role in chronic lymphocytic leukemia (CLL). Here, we designed specific oligonucleotides to target endogenous *miR-17* (antagomiR17). In-vitro administration of antagomiR17 effectively reduced *miR-17* expression and the proliferation of CLL-like MEC-1 cells. When injected in-vivo in tumor generated by the MEC-1 cells in SCID mice, antagomiR17 dramatically reduced tumor growth and significantly increase survival. Altogether, our results provide the rationale for the use of antagomiR17 as a novel potential therapeutic tool in CLL and in other lymphoproliferative disorders where *miR-17* has a driver role in tumor progression.

**Electronic supplementary material:**

The online version of this article (doi:10.1186/s13045-014-0079-z) contains supplementary material, which is available to authorized users.

## Findings

We have recently reported that microRNA from the *miR-17 ~ 92* family may be responsible for the increased proliferation/survival in chronic lymphocytic leukemia (CLL) cells expressing unmutated (UM) *IGHV* genes and with high level of ZAP-70 [1]. In particular, the enforced expression of *miR-17* reduced the expression of the tumor suppressor genes *E2F5*, *TP53INP1*, *TRIM8* and *ZBTB4*, and protected CLL cells from apoptosis [1]. Here, we provide evidences that the abrogation of *miR-17* expression by a specific antagomiR is sufficient to inhibit leukemic growth and progression both in-vitro and in-vivo.

Peripheral blood samples from CLL patients were obtained in accordance with local Institutional Review Board requirements and declaration of Helsinki. CLL cell stimulation, microRNA and gene expression were performed as reported [1,2]. MEC-1 CLL-like cell line was transfected with a molecule against *miR-17* (hereafter antagomiR17), or scrambled control. In in-vivo experiments, tumors generated by MEC-1 cells into severe combined immunodeficiency (SCID) mice were treated with antagomiR17, scrambled control, or saline solution (see Additional file 1).

**Figure 1:**
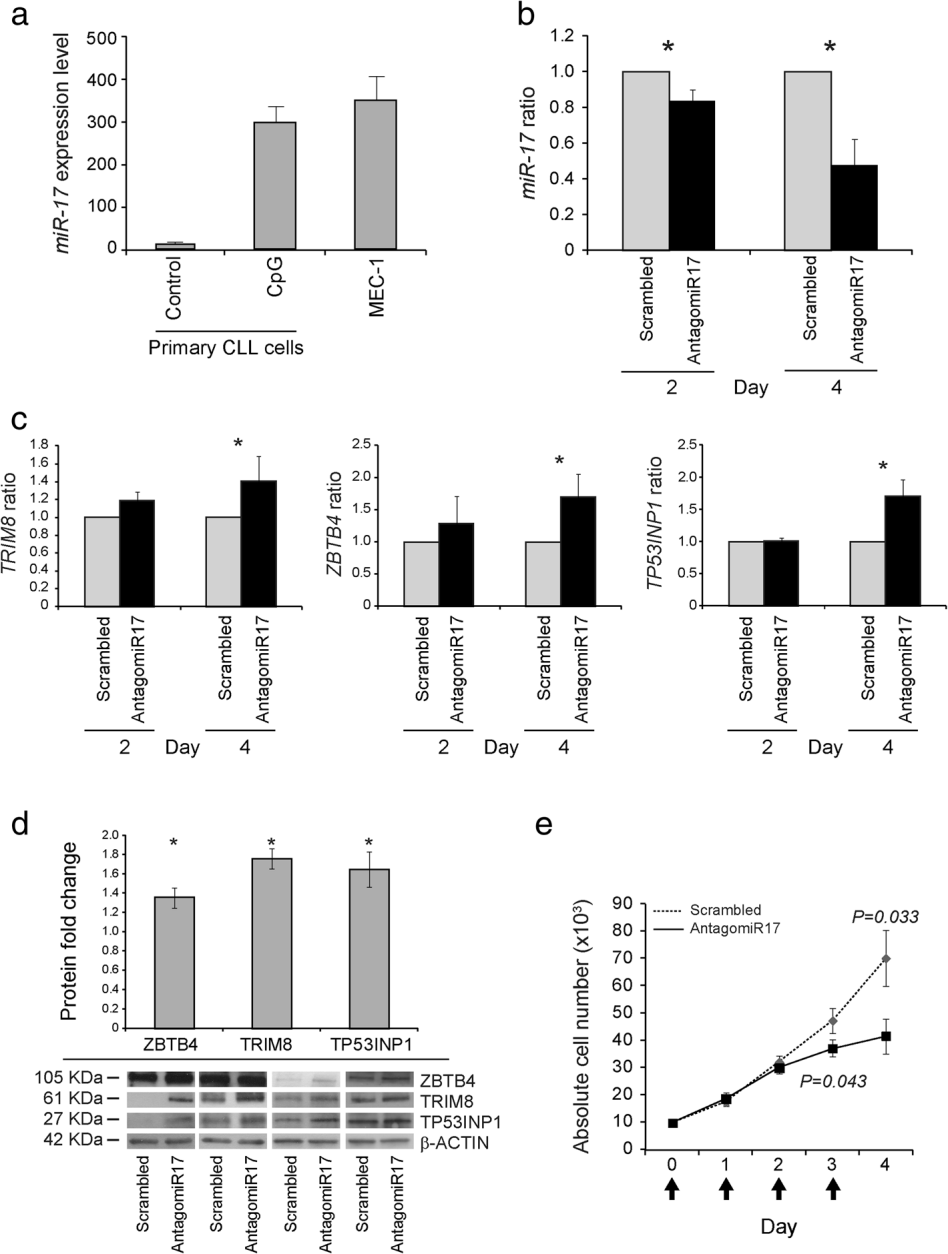
**In-vitro experiments. (a)** *miR-17* expression level in primary UM IGHV CLL cells left unstimulated (control) or stimulated with CpG-ODN (CpG) and in MEC-1 cell lines, as investigated by quantitative real-time polymerase chain reaction (qRT-PCR). Data represent mean ± SEM. **(b)** Expression of *miR-17* in MEC-1 cells transfected with antagomiR17 or scrambled control. *miR-17* expression was evaluated by qRT-PCR at different time-points (2 and 4 days). Data represent mean ± SEM of three replicates. P values (Student’s t-test) for each time-point are shown. *P < 0.05 (antagomiR17 *versus* scrambled control). **(c)** Expression of *TRIM8*, *ZBTB4*, and *TP53INP1* in MEC-1 cells transfected with antagomiR17 or scrambled control. Gene expression was evaluated by qRT-PCR at different time-points (2 and 4 days). Data represent mean ± SEM of three replicates. P values (Student’s t-test) for each time-point are shown. *P < 0.05 (antagomiR17 *versus* scrambled control). **(d)** Effects of antagomiR17 transfection on TP53INP1, TRIM8 and ZBTB4 protein levels in MEC-1 cells. Protein expression levels were measured by Western blot experiment. Lower panel. Relative TP53INP1, TRIM8 and ZBTB4 protein expression levels of MEC-1 cells transfected with antagomiR17 or scrambled control assessed by Western blot. β**-**Actin levels were used as loading control in all cases. Upper panel. In all graphs values are represented as mean fold expression with respect to transfection with scrambled control. Data represent mean ± SEM of four replicates. P values (Student’s t-test) for each time-point are shown. *P < 0.05 (antagomiR17 *versus* scrambled control). **(e)** Proliferation of MEC-1 cells transfected antagomiR17. MEC-1 cells were transfected with antagomiR17 or scrambled control and counted once a day for four days. Dotted line indicates scrambled control transfected cells and solid line indicates antagomiR17 transfected cells. P value (Student’s t-test) is shown. Data represent mean ± SEM of three biological replicates.

The MEC-1 cell line expressed *miR-17* levels comparable to those of CLL samples in which proliferation is triggered by CpG-ODN (Figure 1a). In MEC-1 cells, antagomiR17 transfection significantly reduced *miR-17* expression respect to scrambled control, both at day 2 (mean fold change 0.84 ± 0.06; *P = 0.049*) and at day 4 (mean fold change 0.48 ± 0.14; *P = 0.021*; Figure 1b). Moreover, the *TP53INP1*, *TRIM8* and *ZBTB4* expression showed a significant up-regulation after antagomiR17 treatment both at transcript and protein levels (Figure 1c,d). Finally, MEC-1 cells showed a significant reduction (*P = 0.033*) of cell rate proliferation when transfected with antagomiR17 (Figure 1e). Complementary experiments performed using sorting procedures after transfecting MEC-1 cells with a Cy3-labelled antagomiR17 (Cy3-antagomiR17, Additional file 2: Figure S1a) showed that the Cy3-antagomiR17 bright fraction presented a significant decrease in cell proliferation respect to the Cy3-antagomiR17 dim fraction at day 7 (*P = 0.008*; Additional file 2: Figure S1b). Notably, using a Cy3-labelled scrambled control no difference in MEC-1 cell proliferation was observed (Additional file 2: Figure S1c,d). Altogether, these data demonstrated that antagomiR17 administration effectively reduced the expression of *miR-17* and cell proliferation.

**Figure 2:**
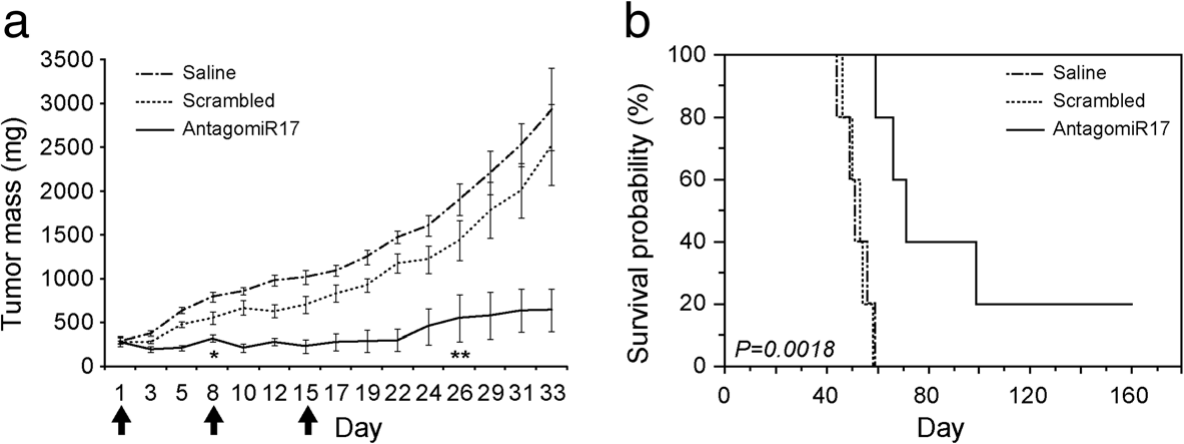
**In-vivo experiments. (a)** Treatment of mice with antagomiR17 inhibits in vivo tumor growth. Plot represents growth curves of tumor-bearing SCID mice treated with either antagomiR17 (5 mice) or scrambled control (5 mice) or saline solution (5 mice) for three times/2 weeks (injections are indicated by arrows). The mass volume of the tumors was measured every two/three days and reported as tumor mass (mg). Dashed, dotted, and solid line indicate mice treated with saline solution, scrambled control, and antagomiR17, respectively. Data represent mean ± s.d. of five biological replicates. *P < 0.05 (antagomiR17 *versus* scrambled control), **P < 0.01 (antagomiR17 *versus* scrambled control). **(b)** AntagomiR17 treatment is associated with increased survival. Kaplan-Meier curves showing percentage of survival probability of tumor-bearing SCID mice treated with antagomiR17 (5 mice) or scrambled control (5 mice) or saline solution (5 mice). Dashed, dotted, and solid line indicate Kaplan-Meier curves of mice treated with saline solution (median OS 52 days), scrambled control (median OS 52 days), and antagomiR17 (median OS 91 days), respectively. The reported P value refers to log-rank test.

Tumors generated by MEC-1 cells injected into SCID mice were treated three times (day 1-8-15) either with antagomiR17 or scrambled control. AntagomiR17 dramatically inhibited tumor growth; this effect, already relevant after the first week of therapy, was maintained till the end of the treatment (Figure 2a) leading to the complete regression of the mass in 1/5 (20%) of cases (not shown). Conversely, administration of the scrambled control resulted in a tumor growth kinetic superimposable to saline-treated tumors (Figure 2a). Of note, a single injection of antagomiR17 was sufficient to significantly reduce tumor growth for at least two weeks after treatment (Additional file 2: Figure S1e). Consistently, median overall survival (OS) of mice treated with antagomiR17 was significantly longer than median OS of mice treated with scrambled control (91 *versus* 52 days, respectively, *P = 0.0018*) or saline solution (91 *versus* 51 days, respectively, *P = 0.0044*) (Figure 2b). Notably, none of the mice showed signs of toxicity. Altogether, these results demonstrate that in-vivo treatment with antagomiR17 significantly abolishes tumor growth and increases survival.

Evidences reported here underline that *miR-17* knockdown is sufficient to block CLL-like cells proliferation both in-vitro and in-vivo. Clinically, despite recent treatment advances, some CLL seem to be refractory to the new drugs [3-5]. In this context, antagomiR treatment may represent a commendable alternative, also considering recent antagomiR phase II trials [6-8]. This strategy could be extended to other lymphoproliferative disorders where *miR-17 ~ 92* amplification and/or overexpression have a pathogenetic role [9,10]. In conclusion, our results highlight the therapeutic potential of antagomiR17, providing the rationale for its use also in the context of specific target delivering systems (e.g. nanoparticles).

## Additional files

Additional file 1:
**Supplemental material and methods.**
Additional file 2: Figure S1. In-vitro control experiments.
