# Supplementary material for: Potential therapeutic role of antagomiR17 for the treatment of chronic lymphocytic leukemia
Source: J Hematol Oncol. 2014 Oct 23;7:79. doi: 10.1186/s13045-014-0079-z (PMC4210490; doi:10.1186/s13045-014-0079-z)
Supplement: Additional file 1: — Supplemental material and methods. [file 13045_2014_79_MOESM1_ESM.docx]

Potential Therapeutic Role of AntagomiR17 for the Treatment of Chronic Lymphocytic Leukemia

**Materials and methods**

**Cell line**

The MEC-1 CLL-like cell line, obtained from DMSZ (<http://www.dsmz.de/>), was cultured according to provider’s guidelines.

**CLL patients**

Peripheral blood (PB) samples from CLL patients were obtained after written informed consent in accordance with local Institutional Review Board requirements (IRB-04-2010, Centro di Riferimento Oncologico, Aviano, Italy) and declaration of Helsinki. The purity of the CLL cells, as monitored by flow-cytometry, always exceeded 95% of clonal CD5^+^/CD19^+^ cells. **All the experiments involving animals were done in compliance with the guidelines of the European (86/609/EEC) and the Italian (D.L.116/92) laws, and were approved by both the Italian Ministry of Health and the Administration of the University Animal House (Prot. 42/2012).**

**Cell culture conditions**

Freshly isolated negatively-selected CLL cells were cultured (1 × 10^7^ cells/ml) in RPMI-1640 supplemented with 10% heat-inactivated fetal bovine serum, 100 U/ml penicillin, 0.1 mg/ml streptomycin, 2 mM L-glutamine and 1 mM sodium pyruvate (Invitrogen, Carlsbad, CA, USA) in the presence or not of 7.5 μg/ml complete phosphorothioate CpG-ODN oligonucleotide 2006 (5′-TCG TCG TTT TGT CGT TTT GTC GTT-3′; Microsynth, Balgach, Switzerland) for 18 hours, as previously reported [1, 2].

**RNA extraction**

Total RNA was extracted using the TRIZOL Reagent (Life Technologies, Carlsbad, CA, USA) and validated for integrity and purity using the Agilent 2100 Bioanalyzer (Agilent Technologies, Santa Clara, CA, USA).

**Quantitative-real-time polymerase chain reactions (qRT-PCR)**

Expression of *miR-17* and of the control *RNU6B* was assessed using a standard TaqMan MicroRNA assay kit (Life Technologies) according to the manufacturer’s instructions and as previously described [3, 4]. Briefly, microRNA was reverse transcribed to cDNA using gene-specific primers and the relative amount of each microRNAs was computed using the equation 2^-ΔCt^, where ΔCt=(Ct _microRNA_ – Ct _RNUB6_).

Expression of specific genes of interest (*TP53INP1, TRIM8, ZBTB4*, and *β_2_*-microglobulin, *β_2_M)* was evaluated with the TaqMan Gene Expression assay kit (Life Technologies); the relative amount of each gene was calculated as above but using expression of *β_2_M* as internal control. Fold-change between classes was calculated as reported [4]. All qRT-PCR experiments were performed on a Bio-Rad Cfx96 (Bio-Rad Laboratories, Hercules, CA, USA).

**Western blot**

Total proteins were extracted from MEC-1 cells collected after antagomiR transfection, loaded and run in 4–15% Mini-PROTEAN TGX Stain-Free gel (Bio-Rad) for Western analysis and detection by ECL (GE Healtcare, UK) or Immobilon (Millipore Corporation, MA). 1:500 rabbit-anti- TP53INP1 (Abcam, Cambridge, MA), 1:1000 rabbit-anti-TRIM8 (Abcam), 1:1000 rabbit-anti-ZBTB4 (Abcam) were used for protein detection. Anti-beta Actin antibody (AC-15) (HRP) (**Abcam, UK**) was used for loading control (final dilution 1:100.000). Densitometric quantitation of western blots was determined with the Quantity One 4.1.0 software (Bio-Rad).

**Transfection**

Twenty ng of antagomiR17 (5’-mC mU mA mC mC mU mG mC mA mC mU mG mU mA mA mG mC mA mC mU mU mU mG-3’) Integrated DNA Technologies, IDT, Coralville, IA, USA), or scrambled control (**5’-mA mU mU mU mC mA mU mG mA mC mU mG mU mU mA mC mU mG mA mC mC mU-3’) (IDT)**, were transfected everyday for four consecutive days into 1 x 10^4^ MEC-1 cells using FuGENE HD Transfection Reagent (Promega, Madison, WI, USA). Cells were counted everyday for four consecutive days. m indicates 2'O-Methyl RNA.

Three μg of Cy3-antagomiR17 (5’- /5Cy3/ mC mU mA mC mC mU mG mC mA mC mU mG mU mA mA mG mC mA mC mU mU mU mG -3’) (IDT) were transfected into 5 x 10^6^ MEC-1 cells with the Amaxa Nucleofector system (Lonza Cologne GmbH, Germany) according to manufacturer’s guidelines. As negative control, cells were transfected with equal amounts of Cy3-scrambled control (**5’- /5Cy3/ mA mU mU mU mC mA mU mG mA mC mU mG mU mU mA mC mU mG mA mC mC mU -3’**) (IDT).

**Cell Sorting**

24 hours after transfection, MEC-1 cells with Cy3-antagomiR17, or Cy3-scrambled molecule, were sorted with a FACSAriaIII (BD Biosciences) using the Cy-3 for detection of dim and bright fractions.

**In-vivo administration of antagomiR17 in tumors generated by MEC-1 cells**

**Female** severe combined immunodeficiency (SCID) **mice (4–6 weeks of age) were purchased from Charles River (Milan, Italy) and maintained under pathogen-free conditions. All the experimental procedures involving animals were done in compliance with the guidelines of the European (86/609/EEC) and the Italian (D.L.116/92) laws, and were approved by both the Italian Ministry of Health and the Administration of the University Animal House (Prot. 42/2012). 1 x 10^7^ MEC-1 cells were subcutaneously injected into the flank of mice. When the tumors reached an average volume of 250-300 mm^3^, the tumor-bearing SCID mice were treated with either antagomiR17, or scrambled control, or saline solution. 300 µg of antagomiR17, or scrambled control (both diluted in 50μl of saline) or saline were added to the same volume of Lipofectamine2000 (Invitrogen, Milan, Italy) and then injected intratumorally. The treatment was repeated three times in 15 days. Tumor size was assessed every two/ three days by caliper measurement. Tumor volume was calculated as follow: volume = D x d^2^ x π/6, where D and d are the longer and the shorter diameters, respectively.**

**References**

1 Longo PG, Laurenti L, Gobessi S, Petlickovski A, Pelosi M, Chiusolo P, Sica S, Leone G, Efremov DG: **The Akt signaling pathway determines the different proliferative capacity of chronic lymphocytic leukemia B-cells from patients with progressive and stable disease**. *Leukemia* 2007, **21:**110-120.

2 Tarnani M, Laurenti L, Longo PG, Piccirillo N, Gobessi S, Mannocci A, Marietti S, Sica S, Leone G, Efremov DG: **The proliferative response to CpG-ODN stimulation predicts PFS, TTT and OS in patients with chronic lymphocytic leukemia**. *Leuk.Res.* 2010, **34:**1189-1194.

3 Bomben R, Gobessi S, Dal BM, Volinia S, Marconi D, Tissino E, Benedetti D, Zucchetto A, Rossi D, Gaidano G, Del PG, Laurenti L, Efremov DG, Gattei V: **The miR-17-92 family regulates the response to Toll-like receptor 9 triggering of CLL cells with unmutated IGHV genes**. *Leukemia* 2012, **26:**1584-1593.

4 Bomben R, Dal-Bo M, Benedetti D, Capello D, Forconi F, Marconi D, Bertoni F, Maffei R, Laurenti L, Rossi D, Del Principe MI, Luciano F, Sozzi E, Cattarossi I, Zucchetto A, Rossi FM, Bulian P, Zucca E, Nicoloso MS, Degan M, Marasca R, Efremov DG, Del PG, Gaidano G, Gattei V: **Expression of mutated IGHV3-23 genes in chronic lymphocytic leukemia identifies a disease subset with peculiar clinical and biological features**. *Clin.Cancer Res.* 2010, **16:**620-628.

**Figure legend**

**Figure S1. In-vitro control experiments.** (a) Cell sorting. 24h after transfection with Cy3-antagomiR17, using the Amaxa Nucleofector system, MEC-1 cells were sorted with a FACSAriaIII using Cy3 for detection of the bright and dim fractions. A representative plot is reported indicating the Cy3 bright and dim sorting windows utilized for subsequent proliferation experiment. All experiments were done in triplicate. (b) Proliferation of MEC-1 cells transfected with Cy3-antagomiR17. Plot represents growth curve of the Cy3-antagomiR17 dim and bright fraction. Dotted line indicates the Cy3-antagomiR17 dim fraction and solid line indicates the Cy3-antagomiR17 bright fraction. P value (Student’s t-test) is shown. Data represent mean ± SEM of three replicates. (c) Cell sorting. 24h after transfection with Cy3-scrambled control. A representative plot is reported indicating the Cy3 bright and dim sorting windows utilized for subsequent proliferation experiment. All experiments were done in triplicate. (d) Proliferation of MEC-1 cells transfected with Cy3-scrambled control. Plot represents growth curve of the Cy3-scrambled control dim and bright fraction. Dotted line indicates the Cy3-scrambled control dim fraction and solid line indicates the Cy3-scrambled control bright fraction. P value (Student’s t-test) is shown. Data represent mean ± SEM of three replicates. **In-vivo control experiments.** (e) Treatment of mice with antagomiR17 inhibits in vivo tumor growth. Plot represents growth curves of **tumor-bearing SCID mice** treated with either antagomiR17 (4 mice) or scrambled control (4 mice) or saline solution (4 mice) (injection is indicated by arrows). The mass volume of the tumors was measured every two/three days and reported as tumor mass (mg). Dashed, dotted, and solid line indicate Kaplan-Meier curves of mice treated with saline solution, scrambled control, and antagomiR17, respectively. Data represent mean ± s.d. of four biological replicates. * *P<0.05* (antagomiR17 *versus* scrambled control).
