# Supplementary figures and images for: Potential therapeutic role of antagomiR17 for the treatment of chronic lymphocytic leukemia
Source: J Hematol Oncol. 2014 Oct 23;7:79. doi: 10.1186/s13045-014-0079-z (PMC4210490; doi:10.1186/s13045-014-0079-z)

Figure S1

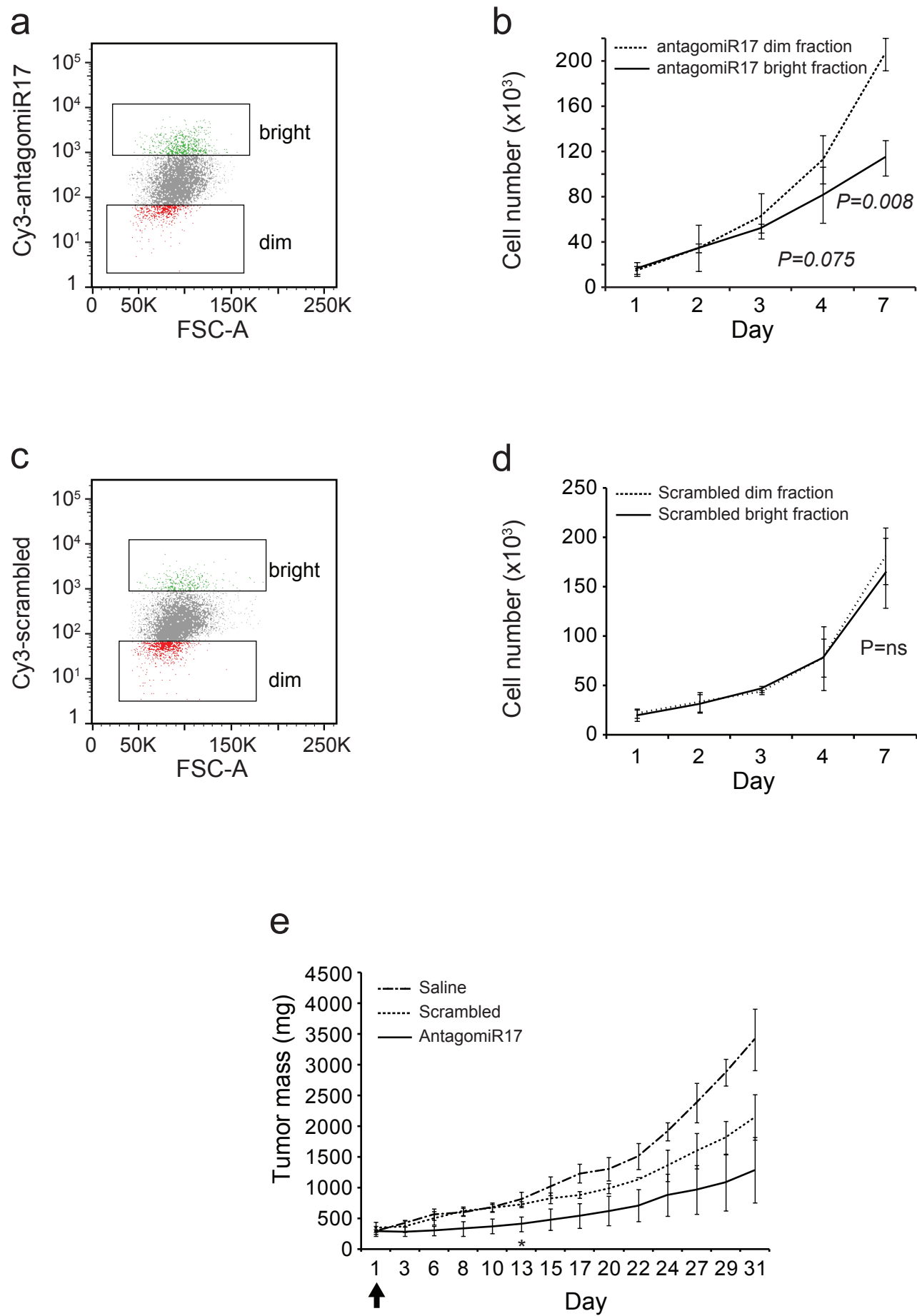

Supplement: Additional file 2: Figure S1. — In-vitro control experiments. [file 13045_2014_79_MOESM2_ESM.pdf]
